# *In situ* Nanoscale Infrared Spectroscopy of Water Adsorption on Nanoislands of Surface‐Anchored Metal‐Organic Frameworks

**DOI:** 10.1002/anie.202011564

**Published:** 2020-11-23

**Authors:** Guusje Delen, Matteo Monai, Florian Meirer, Bert M. Weckhuysen

**Affiliations:** ^1^ Inorganic Chemistry and Catalysis group Debye Institute for Nanomaterials Science Utrecht University Universiteitsweg 99 3584 CG Utrecht The Netherlands

**Keywords:** guest–host interaction, IR spectroscopy, metal-organic frameworks, nano-spectroscopy, SURMOF

## Abstract

Despite technological advancements, probing gas‐solid interfaces at the nanoscale is still a formidable challenge. New nano‐spectroscopic methods are needed to understand the guest–host interactions of functional materials during gas sorption, separation, and conversion. Herein, we introduce *in situ* Photoinduced Force Microscopy (PiFM) to evidence site‐specific interaction between Metal‐Organic Frameworks (MOFs) and water. To this end, we developed amphiphilic Surface‐anchored MOF (SURMOF) model systems using self‐assembly for the side‐by‐side hetero‐growth of nanodomains of hydrophilic HKUST‐1 and hydrophobic ZIF‐8. PiFM was used to probe local uptake kinetics and to show D_2_O sorption isotherms on (defective) HKUST‐1 paddlewheels. By monitoring defect vibrations, we visualized in real‐time the saturation of existing defects and the creation of D_2_O‐induced defects. This work shows the potential of *in situ* PiFM to unravel gas sorption mechanisms and map active sites on functional (MOF) materials.

## Graphical abstract

A new *in situ* nano‐spectroscopy technique elucidating the guest–host chemistry between Metal‐Organic Frameworks (MOFs) and water vapor is described. By using adapted Photoinduced Force Microscopy (PiFM) on amphiphilic surfaces of mixed HKUST‐1/ZIF‐8 films, site‐specific D_2_O gas sorption isotherms could be determined. Furthermore, using *in situ* PiFM, the real‐time evolution of defective HKUST‐1 paddlewheels on the surface could be visualized.

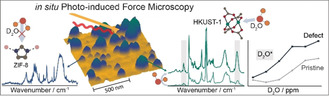

Many chemical and catalytic processes depend on the capture, separation, and conversion of gaseous molecules on functional materials. Such events preferentially occur on specific defective, undercoordinated sites, which can be introduced by rational design to improve material performance.[^1^, ^2^, ^3^] Prominent examples can be found in the booming field of metal‐organic frameworks (MOFs), materials with high porosity, crystallinity, and tunable chemistry which are increasingly finding their way towards applications.[^4^, ^5^] To guide synthetic efforts in this field, and in nanomaterials science in general, it is of paramount importance to move from a bulk to a nanoscale understanding of guest–host interactions.

In surface sciences, efforts have been made to gain a nanoscale understanding of the adsorption of gases on material surfaces. However, these efforts have been dominated by several techniques that work under ultra‐high‐vacuum conditions, far away from real operating conditions, a problem also known as the pressure gap.^[6]^ In contrast, techniques suitable to study adsorbate interaction at elevated pressures, such as vibrational spectroscopy, lack the required spatial resolution to provide structure‐performance relationships of specific (defective) sites.[^7^, ^8^, ^9^] To bridge this gap, new *in situ*, chemical‐ and highly‐sensitive nano‐spectroscopic tools are required.[^8^, ^10^, ^11^]

Our manuscript introduces *in situ* capability to the emerging field of Photoinduced Force Microscopy (PiFM), a highly sensitive and spatially resolved near‐field infrared (IR) technique, to gain new insights into guest–host interactions at working conditions and in real‐time.[^12^, ^13^] We showcase that PiFM can be used to study structure‐dependent adsorption on defective versus non‐defective sites in a MOF by simultaneously uncovering topological and chemical information at the nanoscale with a spatial resolution down to 10 nm.^[13]^ We selected water (D_2_O) as a probe molecule for this proof‐of‐concept because of its omnipresence in industrial gas streams, but highlight that our approach can be extended to many gases/functional materials.[^14^, ^15^]

We have applied *in situ* PiFM to a range of surface‐anchored metal‐organic frameworks (SURMOFs) as is outlined in Figure 1. SURMOFs are well‐known model systems, based on MOF grown on alkanethiol functionalized gold surfaces.[^16^, ^17^, ^18^] Herein, we nanosized the MOF deposits to stimulate defect formation by manipulating the self‐assembly behavior of the thiol molecules promoting/inhibiting MOF growth on the substrates, yielding gapped, and even bifunctional surfaces of HKUST‐1 and ZIF‐8.[^19^, ^20^, ^21^]

**Figure 1:**
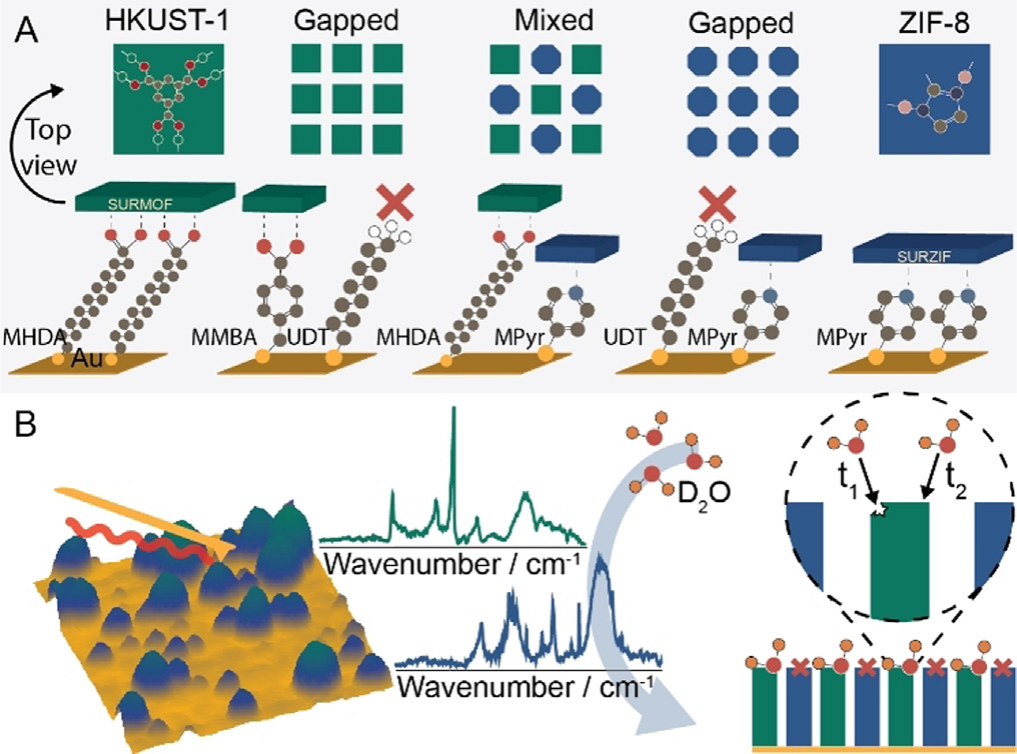
Outline of the used approach. A) Various thiol compositions were used for Au substrate functionalization prior to Layer‐by‐Layer (LbL) synthesis of SURMOF (HKUST‐1, green) and SURZIF (ZIF‐8, blue) and their mixtures. Carboxylic acids selectively promote HKUST‐1 growth, pyridine promotes ZIF‐8 growth, while methyl‐thiols were used to suppress growth. Aromatic thiols were incorporated to create π‐stacked nanodomains leading to either gapped HKUST‐1 or ZIF‐8; or mixed HKUST‐1/ZIF‐8. B) *In situ* Photoinduced Force Microscopy (PiFM) was applied to these SURMOFs, SURZIFs, and related mixtures to deduce their interaction with D_2_O on the nanoscale.

HKUST‐1, an archetypical MOF, is highly hydrophilic and has low hydrothermal stability as its paddlewheel SBU hydrolyses in the presence of water vapor.[^22^, ^23^] On the other hand, Zeolitic‐Imidazolate Frameworks (ZIFs), such as ZIF‐8, are hydrophobic and show increased hydrothermal and chemical stability. By depositing HKUST‐1 and ZIF‐8 together on a substrate, one can create a single amphiphilic, bifunctional surface, displaying two highly distinct water‐MOF/ZIF guest–host interactions.[^24^, ^25^]

Subsequently we studied whether our approach was able to *in situ* detect adsorption of gas molecules on heterogeneous functional surfaces with chemical and nanoscale resolution, and if we could discern site‐specific sorption mechanisms and kinetics. This manuscript goes on to report a combination of isotope labeling and *in situ* nano‐spectroscopy to ultimately show the enhanced uptake of D_2_O on defective HKUST‐1 with undercoordinated Cu sites. Furthermore, we provide direct evidence that water dissociatively adsorbs on these defect sites, and that with increasing pressure, molecularly adsorbed water dissociates on the pristine HKUST‐1 surface thereby forming new defects. This led to the deduction of site‐specific gas sorption isotherms of water on HKUST‐1 and a visual representation of defect formation and propagation as a function of water sorption.

To synthesize nanoislands of SURMOF materials we have used a bottom‐up approach aimed to locally promote or suppress their growth. Using mixed Self‐Assembling Monolayers (SAMs), we have formed nanosized patterns in the thiols used as anchoring sites of SURMOFs. As shown in Figure 1, an aliphatic alkanethiol, 11‐undecanethiol (UDT), was used to suppress SURMOF growth, 4‐(mercaptomethyl)benzoic acid (MMBA), an aromatic thiol with a ‐COOH head group mimicking HKUST‐1 linkers, was used to promote SURMOF growth, while the aromatic 4‐mercaptopyridine (MPyr) was used to promote SURZIF growth. Aromatic thiols were used in combination with the aliphatic UDT as we hypothesized that strong inter‐molecular π‐π interaction would lead to the formation of domains of a substantially larger size than for mixed aliphatic‐aliphatic SAMs (nm vs. Å), which was essential to prevent intergrowth of SURMOF upon Layer‐by‐Layer (LbL) synthesis.^[19]^

The mixed SAM, composed of UDT and MMBA, was first characterized by bulk IR spectroscopy (Figure S1), showing co‐adsorption of both thiols. Atomic Force Microscopy (AFM) analysis showed a nano‐patterned distribution of species on the Au surface, with bimodal height (4; 11 nm) and size (20; 61 nm) distributions, suggesting the formation of segregated domains of UDT and MMBA (Figure 2 A–C). This was confirmed using PiFM, which showed a different IR spectrum on both types of domains (Figure 2 D). For example, the 11 nm thick features possessed a band at 1450 cm^−1^ corresponding to aromatic ring breathing vibrations of MMBA thiols. To visualize the distribution of thiol species, we performed a hyperspectral (hyPIR) measurement consisting of a full IR spectrum on each AFM pixel,^[26]^ in combination with Principal Component Analysis (PCA) and clustering on the acquired data set (Figure 2 C). This analysis allowed for the visualization of domain formation of the chemically distinct thiols over the full AFM surface. Additionally, it showed the aliphatic thiol coverage to be less homogeneous than expected. We propose this to be the result of the disruption of the long‐range Van Der Waals‐stacking by the intermittent π‐stacking domains. Similar findings were made on the mixed MPyr‐MHDA surface (Figure S2). Overall, PiFM was demonstrated to be a nano‐spectroscopy method capable of probing thin films, which were previously impossible to chemically analyze using bulk IR or AFM/STM techniques.

**Figure 2:**
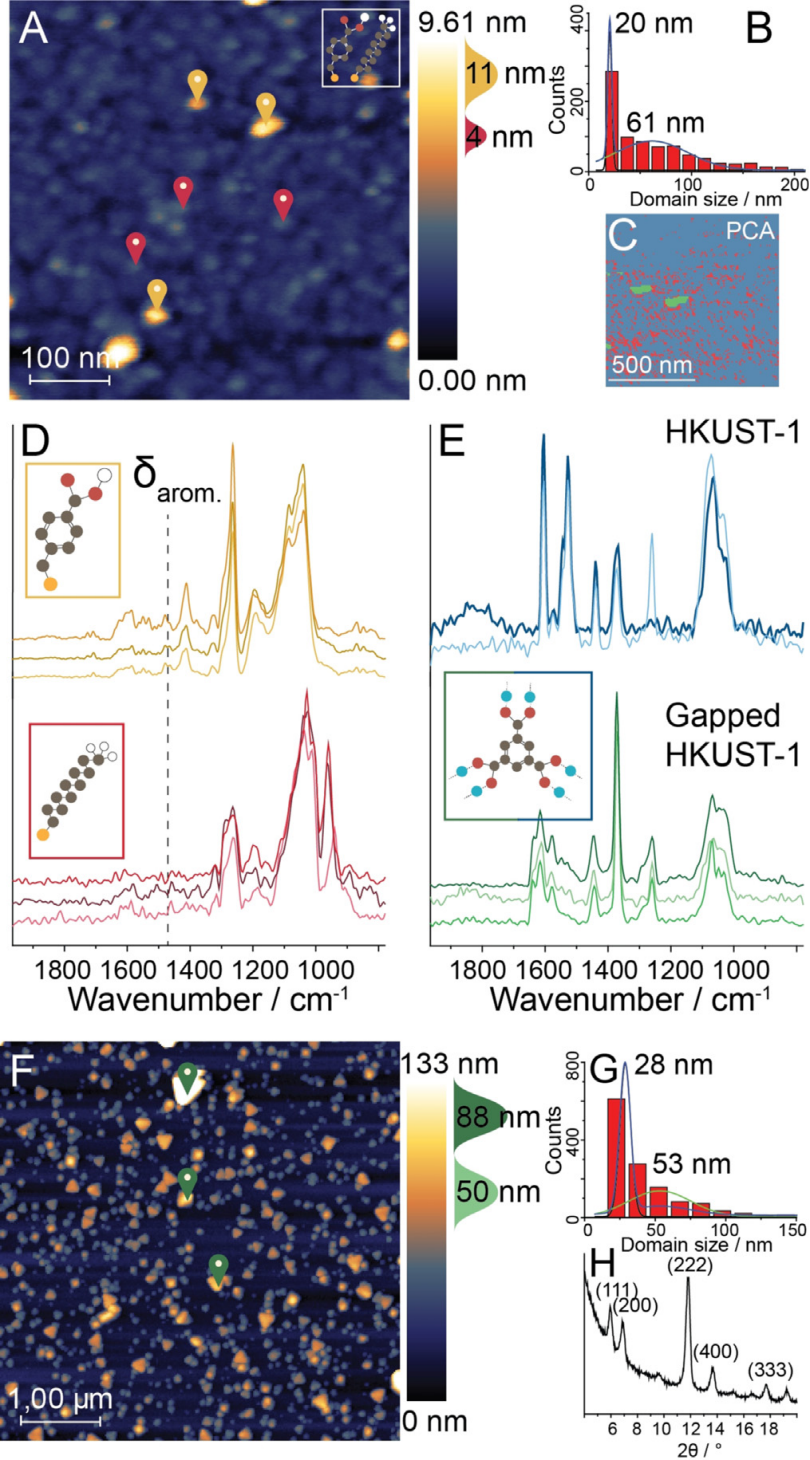
Bottom‐up approach for SURMOF nanosizing as probed by *in situ* Photoinduced Force Microscopy (PiFM). A) AFM image of the MMBA‐UDT surface showing (B) a bimodal height distribution of nm‐sized domains. C) Example of a segmented hyperspectral (hyPIR) image based on Principal Component Analysis (PCA) and clustering. Pixels in the image are pooled based on spectral similarity, showing the thiol distribution (red: UDT, green: MMBA, blue: Au, spectra in Figure S1). D) PiFM point spectra of positions marked in (A). These show aromatic (1450 cm^−1^) vibrations in the MMBA spectrum (top). E) Point spectra of HKUST‐1 grown on MMBA single‐thiol SAM (top) and of the markers in (F) (bottom). F) AFM image of 100 layers of HKUST‐1 grown on the MMBA‐UDT surface showing (G) a gapped SURMOF distribution. F) X‐ray diffractogram showing crystallinity of gapped HKUST‐1.

Subsequently, 100 layers of HKUST‐1 were deposited on the MMBA‐UDT surface (Figure 2 F), showing bulk and PiFM IR spectra comparable to those of bulk HKUST‐1 and HKUST‐1 grown on single thiol surfaces (Figure 2 E, Figure S3).^[27]^ From AFM analysis, we found a bimodal distribution of both height (50; 88 nm) and size (28; 53 nm) of the MOF deposits, indicating two growth regimes in the gapped SURMOF. This can be explained by the results of X‐Ray Diffraction (XRD) (Figure 2 G, H): Whereas HKUST‐1 grown on a single ‐COOH thiol surface is typically preferentially oriented in the [100] direction,^[28]^ the gapped SURMOF displays bulk crystallinity characteristics. This was also visible in the crystal shapes in the AFM images: HKUST‐1 crystals growing in the [100] direction tend to display a triangular shape as opposed to a rectangular shape for the [111] direction.[^29^, ^30^] These triangular crystals are larger, indicating faster growth in the [100] direction. Additionally, the size distributions indicate that HKUST‐1 mainly grows perpendicular to the substrate surface, thereby creating isolated crystals representing bulk crystal properties, making these systems excellent for guest–host interaction studies. The growth of the SURZIF‐8 analog is described in detail in the Supporting Information (Figures S4 and S5).

For the synthesis of the mixed SURMOF (HKUST‐1)/SURZIF (ZIF‐8) surface, 10 layers of both materials were deposited on a mixture of aromatic 4‐mercaptopyridine (MPyr) and aliphatic 16‐mercaptohexadecanoic acid (MHDA). AFM showed MOF/ZIF grains of different morphologies (triangular/hexagonal) and the IR spectra of these grains were chemically distinct: the triangular grains show IR bands typical for HKUST‐1,^[18]^ whereas the octahedral grains show IR bands corresponding to ZIF‐8^[31]^ (Figure 3 B). As PiFM has a probing depth of approximately 30 nm, the results indicate that the two frameworks grew on individual domains, and not on top of the previously grown SURMOF.[^12^, ^32^] Thus, a surface exposing two types of SURMOF, i.e., a bifunctional SURMOF, was synthesized. Macroscopically, the various SURMOFs display a singular bulk characteristic (hydrophilic, hydrophobic, amphiphilic) as shown by rudimentary contact angle measurements (Figure S6). Yet on the nanoscale, to the best of our knowledge, site‐specific behavior is unraveled for the first time by the development of real‐time *in situ* PiFM nano‐spectroscopy (Figure S7).

**Figure 3:**
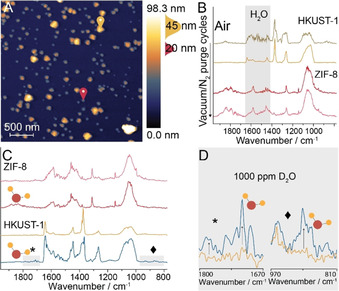
Amphiphilic nature of the bifunctional mixed SURMOF (HKUST‐1)/SURZIF (ZIF‐8) as evaluated with *in situ* Photoinduced Force Microscopy (PiFM). A) AFM image of 10 layers of ZIF‐8/HKUST‐1 grown on MPyr/MHDA. Markers indicate the location of point spectra taken for HKUST‐1 (yellow) and ZIF‐8 (red) before and after vacuum/N_2_ purge cycles. B) IR spectra show the removal of ad‐/physisorbed water on the ambient HKUST‐1 and the water‐independent IR spectrum of ZIF‐8. C) IR spectra of both species after the introduction of 1000 ppm D_2_O, and D) Zoom‐in IR spectra of the two areas marked in (C) showing the appearance of new peaks in the HKUST‐1 spectrum.

Under ambient measurement conditions, the PiFM IR spectra on the HKUST‐1 domains show significant interference from water (Figure 3 B, 1^st^ trace). To remove water, we have performed purging cycles in which we fully evacuated the sample compartment and backfilled it with dry N_2_ (Figure S8). Cycling initially revealed reduced water interference and ultimately exposed the true IR spectrum of HKUST‐1 without water on its surface (Figure 3 B, 2^nd^ trace). In contrast, the PiFM spectrum of ZIF‐8 domains in air showed little water interference (Figure 3 B, 3^rd^ trace), and subsequent vacuum/purge cycling experiments did little to change the IR spectrum of ZIF‐8 (Figure 3 B, 4^th^ trace). To further prove the distinct behavior of the two nanosized domains of the bifunctional film, we backfilled the sample compartment with 1000 ppm D_2_O (Figure 3 C). Whereas this change in the environment did not affect the IR spectrum of ZIF‐8, the IR spectrum of HKUST‐1 presented two new peaks indicating D_2_O adsorption (Figure 3 C, D).

To study the behavior of the hydrophilic SURMOF (HKUST‐1) material in D_2_O vapor in more detail, we have performed hyperspectral (hyPIR) measurements over 5 h starting in N_2_ (post vacuum/N_2_ purging cycles) flowing 3 vol. % D_2_O/N_2_ at 25 mL min^−1^, reaching 9000 ppm in the sample chamber (Figure S7). The D_2_O concentration‐dependent spectra resulting from the hyPIR measurements are plotted in Figure 4 A. These show the appearance and intensity increase of two IR bands over time at 890 and 1725 cm^−1^ (gray boxes), corresponding to molecularly adsorbed D_2_O and deuterated carboxylic acid, respectively, indicating clear guest–host interactions.[^33^, ^34^] These findings correspond to previous reports that have shown that HKUST‐1 provides two types of sites for water sorption, namely the paddlewheel's axial coordinatively unsaturated sites for the molecular sorption of water, and the paddlewheel's equatorial sites for dissociative sorption of water leading to the breaking of the metal‐carboxylate bond and the formation of a paddlewheel defect.[^22^, ^34^, ^35^] To study this guest–host interaction further, we have determined the ratios of several IR bands over time (Figures 4 B–D).

**Figure 4:**
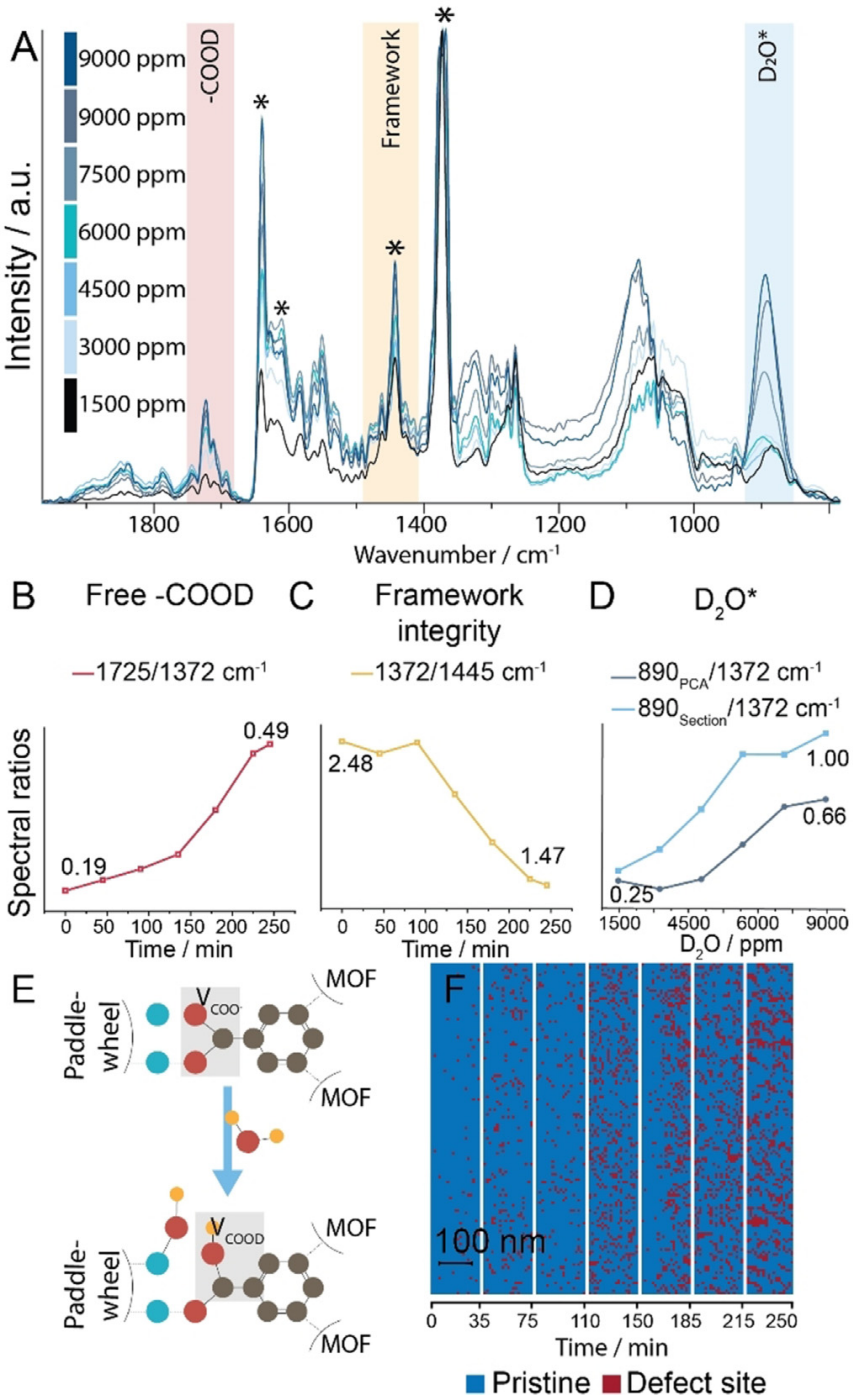
*In situ* Photoinduced Force Microscopy (PiFM) of D_2_O sorption on SURMOF HKUST‐1. A) *In situ* PiFM spectra during D_2_O sorption with increasing D_2_O concentration. Clear changes in the IR absorptions were observed in the regions indicated by colored boxes. Peak ratios of spectra in (A) show B) creation of free deuterated carboxylic acid, as well as C) framework destruction, and D) D_2_O sorption isotherms for (bottom) averaged HKUST‐1 and (top) defective paddlewheel sites. E) Proposed mechanism of defect formation upon D_2_O adsorption on HKUST‐1. F) Spatiotemporal visualization of defect formation (indicated by red, 1 s/pixel) using principal component analysis (PCA) and clustering of time‐based sections of hyperspectral (hyPIR) images upon D_2_O sorption.

The ratio plotted in Figure 4 D (dark blue) represents the average gas sorption isotherm of D_2_O on the SURMOF surface. However, since adsorption on defects is expected to be favored, PCA and clustering were performed on the *in situ* hyPIR data to cluster spectra by spectral similarity, that is, chemical variation of the host (Figure S9). Of the spectra of these clusters, we have calculated the ratio between a known defect vibration and the pristine paddlewheel vibration.^[18]^ This indicated that surface regions showing a higher concentration of defects show a faster response to the D_2_O. To further study D_2_O sorption on defects and to allow for the comparison of site‐specific behavior as a function of D_2_O concentration, we divided the hyPIR measurement into time‐based sections and clustered these sections. In each section, the 890/1372 cm^−1^ ratio was calculated for the clusters showing the highest defect concentration (Figure 4 D, light blue). As a result, Figure 4 D shows the site‐specific gas isotherms displaying the difference in uptake kinetics between the average HKUST‐1 surface and the nanoscale regions of defect‐rich HKUST‐1.

Additionally, we have studied the effect of D_2_O adsorption on the integrity of the HKUST‐1 framework. We observed the creation of free deuterated carboxylic acid over time (Figure 4 B), which suggested the hydrolysis of HKUST‐1 paddlewheels made up of coordinated acids (Figure 4 E). This is in line with mechanisms previously proposed in literature.[^22^, ^34^] However, using our novel *in situ* PiFM approach in combination with isotope labelling we were able to provide direct (nanoscale) evidence for this mechanism. To further study paddlewheel hydrolysis, we calculated the ratio between the aromatic breathing vibration and the coordinated acid vibration, which showed a clear trend of framework destruction upon D_2_O sorption (Figure 4 C). As we hypothesized that this framework destruction led to the formation of new defects, we have used PCA and clustering to map the location of defect‐rich areas over the SURMOF surface, resulting in visual confirmation of the creation of faulty paddlewheels as a result of (heavy) water adsorption (Figure 4 F). Furthermore, quantification of this analysis showed an approximate increase in defect density from 2 % to 25 % (Table S1), whereas inspection of the sections additionally indicated the non‐random formation of propagated defects.^[36]^ Notably, this disintegration of the HKUST‐1 paddlewheels did not lead to extended morphological change, underlining the importance of (nano‐)spectroscopy in guest–host interaction studies (Figure S10).

To conclude, we have demonstrated the use of *in situ* nanoscale IR spectroscopy to investigate guest–host interactions within MOF and ZIF thin‐films. We highlighted the sensitivity of the developed method by mapping mixed thiols SAMs, which were used as templates for SURMOF and SURZIF growth through the layer‐by‐layer synthesis. The resulting amphiphilic HKUST‐1/ZIF‐8 surface was analyzed to reveal individual nanometer‐sized hydrophilic and hydrophobic domains. Furthermore, the hydrophilic HKUST‐1 material was studied to show enhanced D_2_O sorption on existing defective paddlewheel sites, as well as D_2_O induced defect formation in real‐time. Future research will focus on expanding the number of probe gases applied to *in situ* PiFM, such as NO and CO, for gas sorption, and ultimately reactive gases for catalysis research on the nanoscale.

## Conflict of interest

The authors declare no conflict of interest.

## Supporting information

As a service to our authors and readers, this journal provides supporting information supplied by the authors. Such materials are peer reviewed and may be re‐organized for online delivery, but are not copy‐edited or typeset. Technical support issues arising from supporting information (other than missing files) should be addressed to the authors.

Supplementary
