# Supplementary material for: In situ Nanoscale Infrared Spectroscopy of Water Adsorption on Nanoislands of Surface‐Anchored Metal‐Organic Frameworks
Source: Angew Chem Int Ed Engl. 2020 Nov 23;60(3):1620–4. doi: 10.1002/anie.202011564 (PMC7839449; doi:10.1002/anie.202011564)
Supplement: Supplementary file 1 — Supplementary [file ANIE-60-1620-s001.pdf]

Angewandte
Eine Zeitschrift der Gesellschaft Deutscher Chemiker
Chemie

Supporting Information

In situ Nanoscale Infrared Spectroscopy of Water Adsorption on Nanoislands of Surface-Anchored Metal-Organic Frameworks

*Guusje Delen, Matteo Monai, Florian Meirer, and Bert M. Weckhuysen**

anie_202011564_sm_miscellaneous_information.pdf

Supporting Information

Table of Contents

Materials and Methods

Extended Analysis of Thiol Domain Formation

Figure S1 GI-ATR-IR spectrum of co-adsorbed aromatic MMBA and aliphatic UDT thiol.

Figure S2 PiFM measurements on (mixed SURMOF/-ZIF grown on) a mixed MHDA-MPyr thiol surface.

Figure S3 Analysis of 100 layers of HKUST-1 grown on MMBA.

Figure S4 Analysis of 100 layers of ZIF-8 grown on MPyr.

Figure S5 AFM analysis of 100 layers gapped SURZIF-8.

Figure S6 Rudimentary water contact angle measurements of SURMOF and SURZIF.

Figure S7 Experimental setup of *in situ* Photo-induced Force Microscopy (PiFM) measurements.

Figure S8 PiFM spectra showing water removal through vacuum/N_2 purge cycles.

Figure S9 Principal component analysis (PCA) of *in situ* PiFM measurement.

Table S1 Defect concentrations of surfaces in hyPIR sections during D_2O sorption.

Figure S10 AFM images of HKUST-1 pre- and post- D_2O adsorption.

Figure S11 Grazing-Incidence Attenuated Total Reflection Infra-Red (GI-ATR-IR) spectra of aliphatic and aromatic thiols.

Figure S12 Atomic Force Microscopy (AFM) images of thiols possessing a carboxylic acid headgroup.

Materials and Methods

Chemicals and Materials. The following chemicals were used: 16-mercaptohexadecanoic acid (MHDA, 99%, Sigma-Aldrich), 4-mercaptobenzoic acid (MBA, 99%, Sigma-Aldrich), 11-undecanethiol (UDT, 98%, Sigma-Aldrich), 4-(mercaptomethyl)benzoic acid (MMBA, 98+%, Sigma-Aldrich), 4-mercaptopyridine (MPyr, 95%, Sigma-Aldrich), ethanol (99.5%, Acros), copper(II) nitrate trihydrate (99+%, Sigma-Aldrich), trimesic acid (95%, Aldrich), zinc(II) nitrate hexahydrate (98%, Sigma-Aldrich), 2-methylimidazole (99%, Sigma-Aldrich), methanol (99.9%, Sigma-Aldrich). Substrates of 60 nm Au on Si, with a 5 nm Ge adhesion layer were provided by AMOLF.

Substrate Preparation. Gold wafers were cleaned in three successive steps. They were rinsed with ethanol, dried in flowing N_2 (4 bar), and cleaned using UV-ozone for 20 min. The clean substrates were immersed in a 1 mM ethanolic SAM solution for 48 h. When 1:1 mixtures of SAMs were used in solution, the total concentration was kept at 1 mM. After functionalization, substrates were rinsed in flowing ethanol before drying with N_2 (4 bar).

MOF Deposition. HKUST-1 SURMOF was synthesized according to our previously described protocol[1]. ZIF-8 Layer-by-Layer (LbL) parameters for the Successive Ionic Layer Adsorption and Reaction (SILAR) setup were: a dipping time of 2 min in both the methanolic metal- (10 mM) and linker (20 mM) solution, respectively. After dipping, the substrate was stirred in methanol for 3 s at 150 rpm.

X-Ray Diffraction (XRD) was performed on a Bruker D8 in grazing incidence diffraction geometry. XRD patterns were recorded in a 2Θ range of 4-20 degrees, the incident angle was 0.3 degrees. A Cu source was used.

Grazing-Incidence Attenuated Total Reflection (GI-ATR) measurements were performed using a grazing angle ATR VeeMAX 2 accessory (Pike technologies) in a PerkinElmer Spectrum 1 spectrometer with an MCT detector. The Ge crystal had a set angle of 65 degrees. Spectra were acquired by averaging 50 spectra in the range 4000-650 cm^{-1} with a resolution of 4 cm^{-1}. The IR beam was vertically polarized, the incident angle was 80 degrees, the spectrometer (optics and sample compartment) was purged with N_2 before measurements. Clean gold wafers were used as a background.

Photo-induced Force Microscopy (PiFM) measurements were performed using a VistaScope from Molecular Vista, Inc. NCHAu25-W AFM tips (Force constant 10-130 N/m, Resonance frequency > 320 kHz) coated with 25-70 nm of gold by Molecular Vista were used in dynamic non-contact mode. A driving amplitude of 2 nm was used, and a frequency sweep of the cantilever was performed prior to measurements to determine cantilever resonance frequencies. After this sweep, detection of topography was set to the second mechanical eigenmode resonance (f_1), and photo-induced signal detection was set to the first mechanical eigenmode resonance (f_0). Determination of the first mechanical mode was repeated after approaching the tip to the surface. By using independent eigenmodes, topography and PiFM signal could be detected simultaneously. Using a cantilever setpoint between 80-85%, 256x256 pixel topography images of varying sizes were collected. The midIR source was a Block Engineering tunable quantum cascade laser (QCL) providing a working range of 1965-785 cm^{-1}. Averaged IR spectra were taken in sideband mode (pulse modulation: $f_m=f_1-f_0$) with a pulse duration of 32 ns, and with a spectral resolution of 1 cm^{-1}. Power levels (iris set between 55-90°, <1 mV of most intense peak during engaged first eigenmode frequency sweep) and acquisition times (between 0.1-1 sec) were varied between measurements in such a way that with minimal power, sufficient signal was collected (>300 µV of most intense peak in engaged first eigenmode frequency sweep). The approximate lateral resolution of IR spectra was <10 nm. PiFM data was analyzed using SurfaceWorks software.

Hyperspectral Photo-induced Infrared (hyPIR) images of 128x128 pixels were measured *ex situ* and *in situ*. Scanning speeds were adapted for spectrum acquisition times. For the *in situ* measurements, a vacuum cell was placed over the AFM head and sample stage. A vacuum pump and a three-way valve with lines to dry N_2, and N_2 flowing through a D_2O saturator were connected. Prior to *in situ* measurements, the vacuum cell was evacuated and backfilled with dry N_2 three times. *In situ* hyPIR measurements were set for 5 h. The measurement started in N_2 and gradually D_2O was introduced. We flowed 3 vol.% D_2O/N_2 at 25 mL/min, reaching 9000 ppm in the sample chamber.

Principal Component Analysis (PCA) and k-means clustering was used to analyze the hyPIR images. Prior to PCA and clustering, the data was normalized by sum image division. In the TXM-XANES-Wizard[2], the data was mean-centered prior to PCA and k-means clustering. The appropriate number N of principal components (PCs) was

selected upon inspection of scree plot, eigenspectra, and eigenimages. K-means clustering was then performed in N-dimensional PC space using twice the number of clusters (2 x N). The result of k-means clustering was subsequently refined gaussian mixture modeling (GMM) using expectation maximization (EM). This provided a class membership value (between 0 and 1) of each pixel to each cluster. The spectrum of each cluster was then determined as the weighted average of all pixels using this class membership. This method thus efficiently pools pixels based on spectral similarity (and not on spatial correlation) and provides excellent estimates for the most different spectra present in the data. The *in situ* hyPIR images were divided into 7 time-based domains. Of each of these sections, the averaged IR spectrum was used to describe average D_2O sorption over time/D_2O concentration. These spectra were used to construct Fig. 4A-D. Each of these sections was clustered using PCA to study the D_2O sorption behavior on defect sites at known D_2O concentrations. These clustered sections were used to construct Fig. 4B, F. More information can be found in Fig. S9.

Extended Analysis of Thiol Domain Formation

Literature has reported that alkanethiols with alternate chain length or functional groups can co-adsorb on Au[3,4]. Unequal thiol solubility is mirrored in the strength of the Van der Waals (VdW) forces exerted between the molecules leading to domain formation[5]. These domains in the range of tens of Ångstroms were visualized using scanning tunneling microscopy (STM) techniques[3,6]. However, in order to use mixed alkanethiol surfaces for SURMOF preparation, this domain size needs to be increased. Otherwise, the intergrowth of MOF pillars into a single slab will negate the SURMOF nanosizing effects. Therefore, the question is whether we can introduce and visualize an alternate stacking effect to create larger thiol domains that are able to promote pillared SURMOF growth in the 10s of nanometers range, thereby making nanosized SURMOF model systems.

To achieve this goal, we propose using thiols with aromatic backbones instead of aliphatic chains, as we hypothesize that their strong inter-molecular π-π interaction will lead to stacking and help to form domains of a substantially larger size. If these larger aromatic thiol domains form, then it is possible to show them using AFM measurements instead of the more demanding STM measurements[7,8]. However, to verify that the aromaticity is responsible for forming larger domains, chemical identification of the domains is vital[9]. In earlier studies, far-field infrared spectroscopy was applied to characterize the functionalized gold surfaces, however, this was only able to measure averaged information of mixed compound deposition[6,10,11]. In this work, we show that recent progress in near-field infrared spectroscopy has made it possible to spectroscopically study the thiol domains beyond the diffraction limit[12]. Before combining aliphatic and aromatic thiols, it was relevant to know whether the aromatic thiol compounds chemisorb in a similar fashion to alkanethiols. We used Grazing-Incidence Attenuated Total Reflection Infra-Red (GI-ATR-IR) spectroscopy to show that both types of -COOH thiols can chemisorb on a Au surface by measuring their averaged surface (i.e. bulk) IR spectrum (Fig. S11). Additionally, AFM measurements were performed to study their stacking behavior (Fig. S12).

It is known from the literature that aliphatic thiols do not stack perpendicularly on the Au surface, but rather twist their backbone to form a (mono)layer with an angle of 30 degrees relative to the surface normal[13]. Mercaptobenzoic acid (MBA), however, which has the benzene group directly attached to the thiol group, does not possess this flexibility. As a result, the thiol stacks either perpendicular or, as we suspect based on AFM measurements, parallel to the surface (Fig. S12)[14]. To achieve the required aromatic flexibility an additional carbon atom was inserted into the backbone between the thiol and benzene. The AFM image in Fig. S12 of 4-mercaptomethyl)benzoic acid (MMBA) functionalized surface showed significantly reduced roughness.

To test whether the use of MMBA resulted in accessible SURMOF nucleation points, we performed an HKUST-1 LbL synthesis according to our previously reported procedure (Fig. S3)[1]. GI-ATR-IR showed the distinct features of Cu-BTC coordination, such as the Cu-coordinated asymmetric and symmetric carboxylate stretching vibrations at 1650 and 1377 cm^{-1}, respectively [1,15]. The formation of the [100] oriented, crystalline HKUST-1 SURMOF was confirmed by its XRD diffractogram [1]. This showed that the orienting effect observed for aliphatic thiols with a carboxylic headgroup remained present for aromatic MMBA thiols.

Despite the successful implementation of a modified aromatic thiol as SURMOF nucleation layer, the question remains whether domains of growth-promoting aromatic thiol can be formed when mixed with a growth-passivating aliphatic thiol. To study domain formation an Au surface was functionalized with an MMBA-UDT (11-UnDecaneThiol) mixture. The co-adsorption of both compounds was verified by bulk IR (Fig. S1). Interestingly, we observed a diminished dimer IR signal for the MMBA thiols which suggested the successful side-by-side

chemisorption of the thiols. Using AFM, we have measured a bimodal height distribution (4; 11 nm) on the surface (Fig. 2A). Even though the UDT thiol has a longer backbone and therefore should be the taller species on the surface, the previously observed stacking behavior of the aromatic thiols casts doubt on which average height corresponds to which thiol. To identify the (assumed distinct) chemical identities, we utilized an AFM-based near-field IR spectroscopy tool, namely Photo-induced Force Microscopy (PiFM), which was able to acquire IR information beyond the diffraction limit, down to the nm scale.

With the PiFM at hand, we were able to measure the IR spectrum of 2 different species on the MMBA-UDT surface (Fig. 2B). This showed that the spectrum of the higher feature possessed a band at 1450 cm^{-1} indicating aromatic ring breathing vibrations. So, from these spectra, we deduced that the higher features -which also showed larger domain areas (Fig. 2C)- were the aromatic species.

By combining two thiols with distinct chemistries we effectively covered the surface with two chemical functionalities, namely with SURMOF growth-promoting thiols and growth-passivating thiols. Following the same procedure, one could also aim to create a surface where both thiols are growth-promoting, yet for different SURMOF species. If successful this would create a bifunctional SURMOF surface. In order for this to work, a combination of thiols would need to be chosen in such a manner that each thiol promotes the growth of only one SURMOF. Another MOF reported (and shown in Fig. S4) to be SURMOF compatible is ZIF-8[16,17]. This MOF is typically grown on -OH terminated thiols, yet this would be an unsuitable combination as HKUST-1 also grows on -OH groups [18]. However, an inspection of the 2-methylimadazole linker structure suggests that an aromatic pyridine thiol should be highly effective for SURZIF-8 growth. Therefore, a combination between 4-mercaptopyridine (MPyr) and MHDA was chosen for the synthesis of a mixed SURMOF[19,20]. PiFM analysis of the MPyr-MHDA covered surface showed distinct chemical identities, as well as similar aromatic thiol domain areas and surface roughness to the MMBA-UDT surface (Fig. S2). The distribution of species over the surface was visualized using PCA calculations based on chemical similarities between spectra. Some, but less than for MBA, aggregation of MPyr was observed, which can be ascribed to the varying degree of ring strain experienced by both thiols. These results underline that for the thiol mixes reported in this work, π-stacking, rather than headgroup-based solubility, was the main force creating the larger domains.

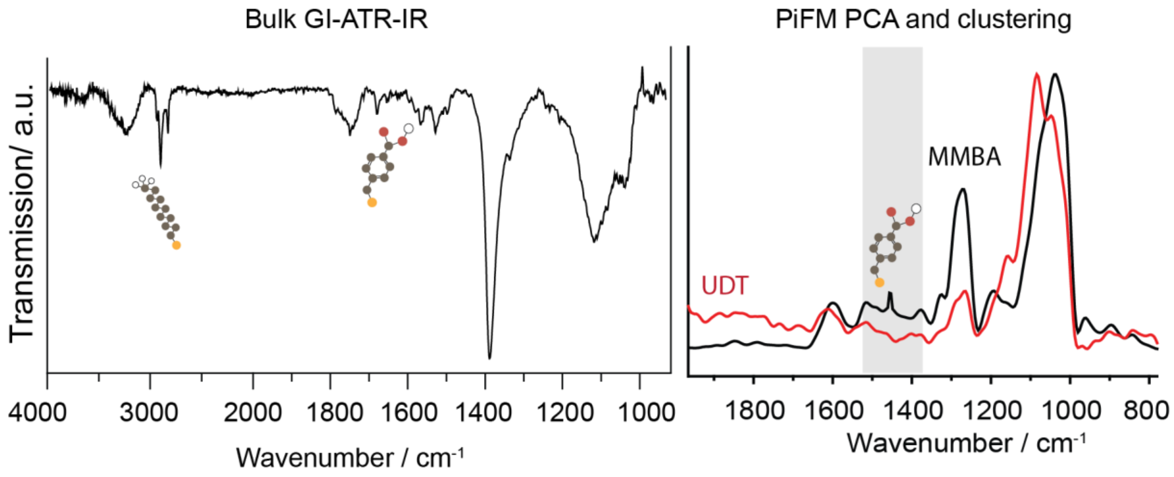

Figure S1: Bulk GI-ATR-IR spectrum (left) and nano-spectra (right) of co-adsorbed thiols 4-(mercaptomethyl)benzoic acid (MMBA) and 1-undecanethiol (UDT). Spectra on the right are the result of averaged spectra of hyperspectral features filtered out by principal component analysis (PCA) and clustering. Nano-spectra belong to image shown in Fig. 2D.

The IR spectrum shows the co-adsorption of the aliphatic (ν_{C-H}=2900, 2950 cm^{-1}) UDT and aromatic MMBA thiols. The $C=O_{monomer}$ vibration at 1750 cm^{-1} is visible and appears to be positively influenced by the presence of the aliphatic thiols, indicating successful side-by-side adsorption of the thiols.

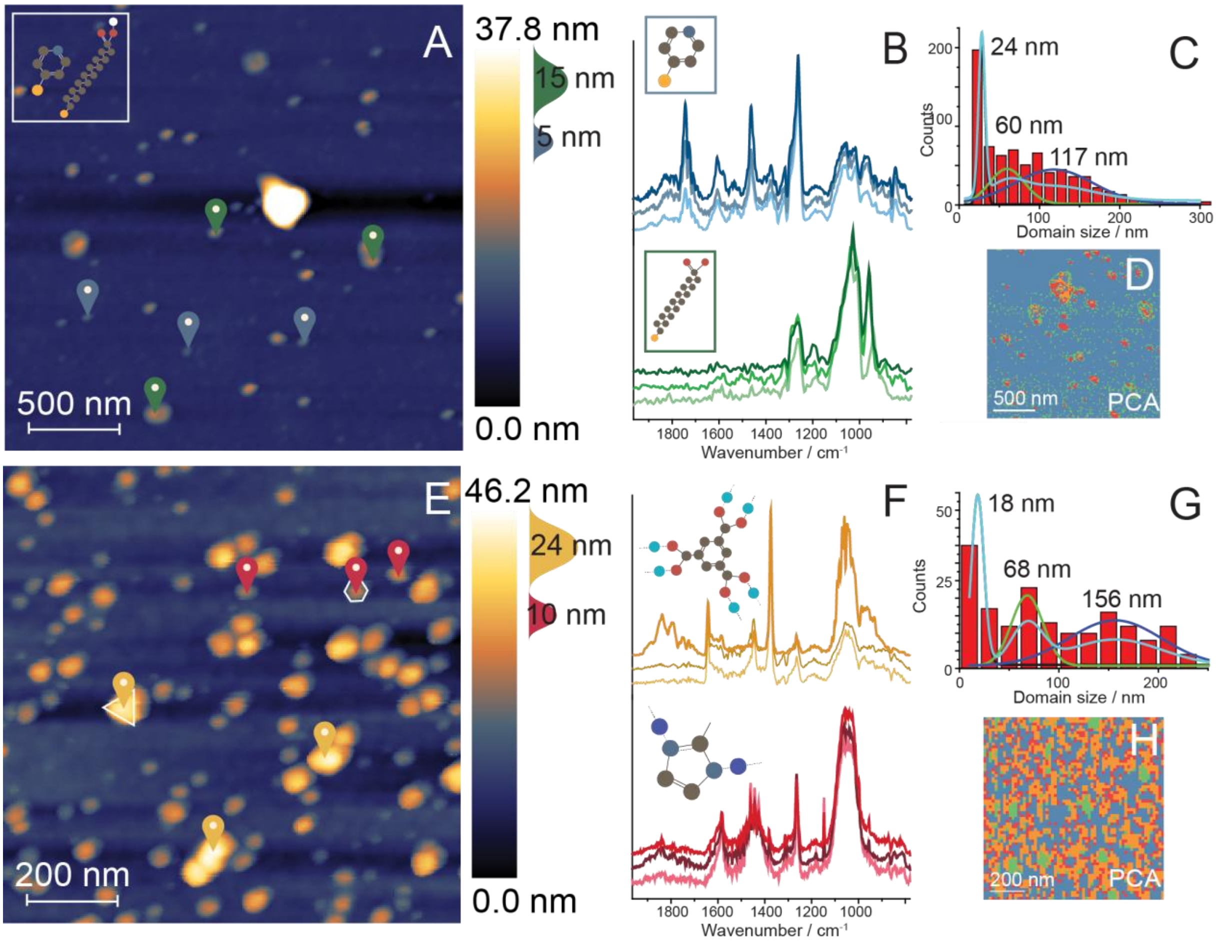

Figure S2: PiFM measurements on (mixed SURMOF grown on) mixed thiol surfaces. A: AFM image of the MPyr-MHDA surface. B: PiFM spectra corresponding to markers shown in (A). C: Domain size distribution of the two thiols shows the creation of domains and some thiol aggregation. D: PCA and clustering analysis showing the distribution of the aromatic (red), aggregated aromatic (orange), and aliphatic (green) species on the surface. E: AFM image of ZIF-8/HKUST-1 grown on MPyr/MHDA. Two grain morphologies suggest the presence of two SURMOFs. F: PiFM spectra corresponding to markers shown in E. The spectra show features typical for HKUST-1 (top) and ZIF-8 (bottom). G: Domain size distribution showing SURMOF areas similar to thiol areas in (C). H: PCA and clustering analysis showing the distribution of SUR-HKUST-1 and SURZIF-8

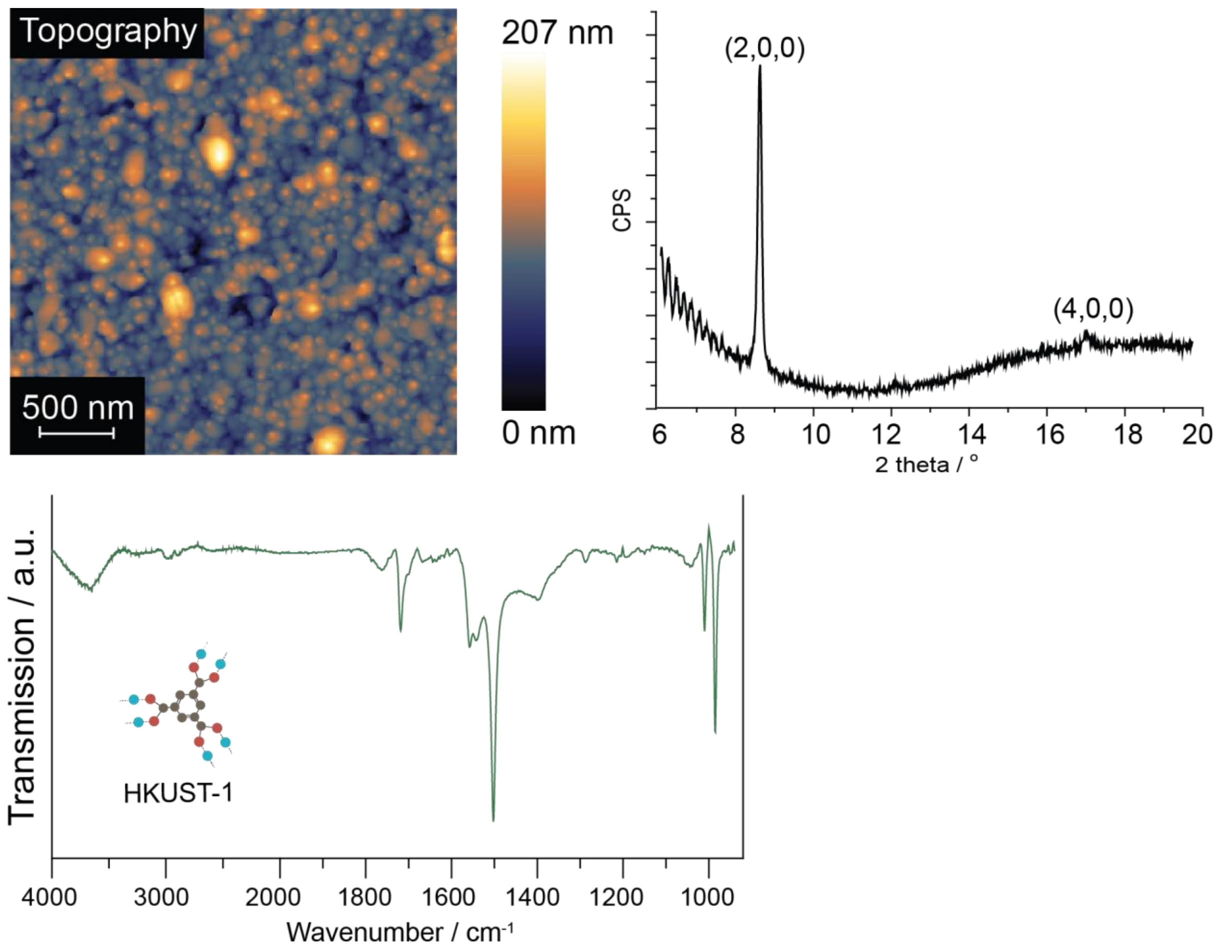

Figure S3: 100 layers of HKUST-1 grown on an MMBA surface. The AFM image shows a fully covered surface of HKUST-1. As a result of the -COOH headgroups of the SAM, oriented crystal growth was observed in the X-ray diffractogram. The bulk IR spectrum measured with GI-ATR-IR displayed peaks typical for HKUST-1.

The AFM image shows the full coverage of the surface with HKUST-1 SURMOF, hence the MMBA thiol film quality is comparable to MHDA and the MMBA thiol is capable of promoting HKUST-1 growth. Film thickness is comparable to previously reported work on HKUST-1 SURMOF on MHDA.[1] The XRD pattern shows the [100] oriented crystallinity of the SURMOF, showing that the orienting effect of the MMBA thiol is unchanged compared to aliphatic -COOH thiols. The averaged IR spectrum showed features typical for HKUST-1.

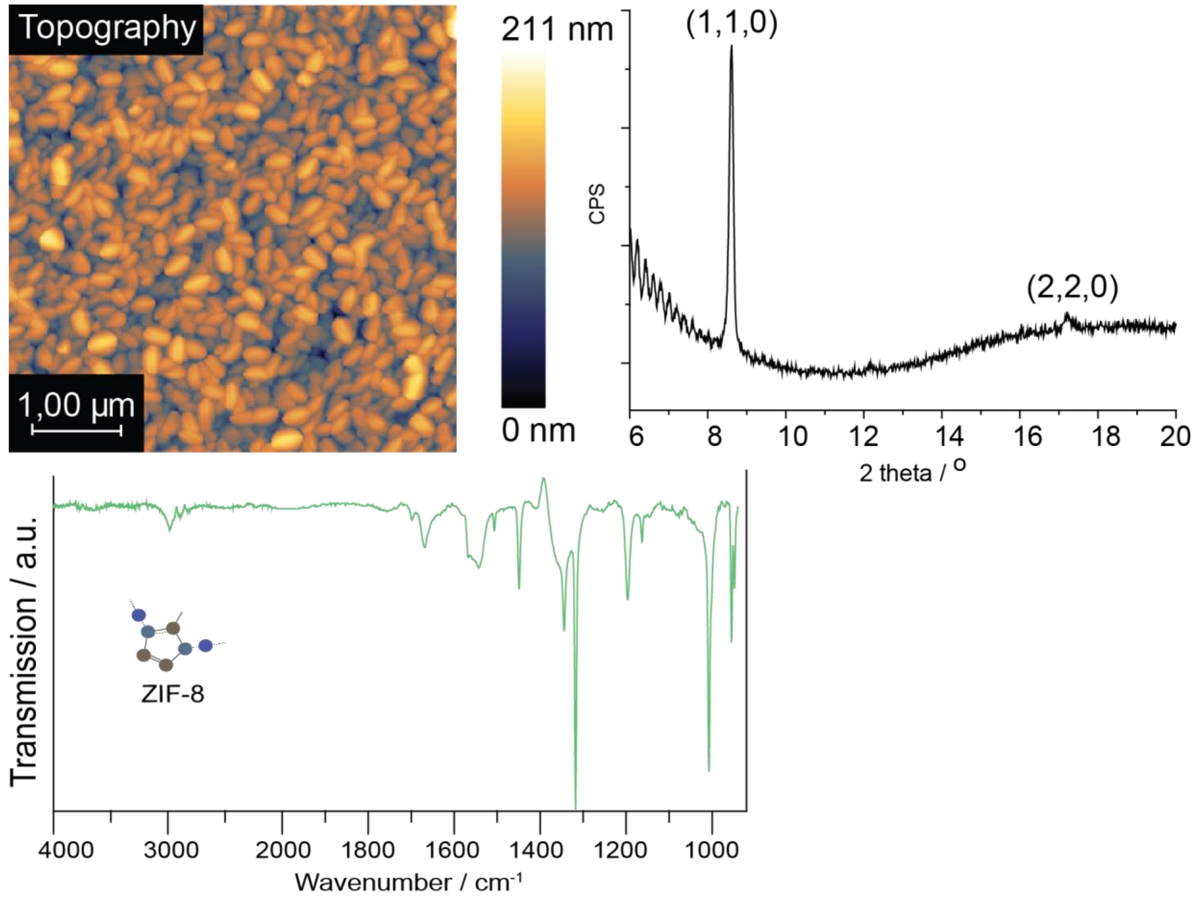

Figure S4: 100 layers of ZIF-8 grown on a MPyr surface. The AFM image shows a fully covered surface of ZIF-8. As a result of the pyridine headgroups of the SAM, oriented crystal growth was observed in the X-ray diffractogram. The bulk IR spectrum measured with GI-ATR-IR displayed peaks typical for ZIF-8.

The AFM image shows a surface fully covered by ZIF-8 grains. The film thickness is comparable to 100 layers of HKUST-1. Thus, MPyr can successfully be used to form a (semi-)monolayer and to promote the growth of ZIF-8 SURMOF. Additionally, the X-ray diffractogram shows that the synthesized ZIF-8 was oriented in the [110] direction, which is the same direction -OH thiols would promote.[17] The bulk IR spectrum showed the chemical distinctness between ZIF-8 and HKUST-1, making them an excellent PiFM case study for bifunctional SURMOFs.

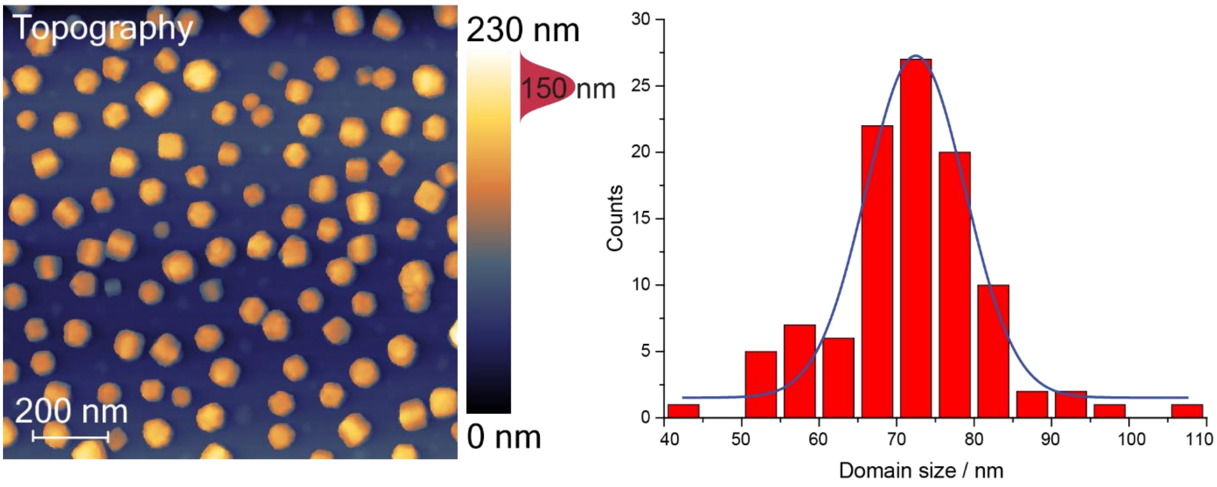

Figure S5: AFM image showing the deposition of 100 layers of gapped SURZIF-8. Particle height and size analysis show well-isolated nano-sized SURZIF growth.

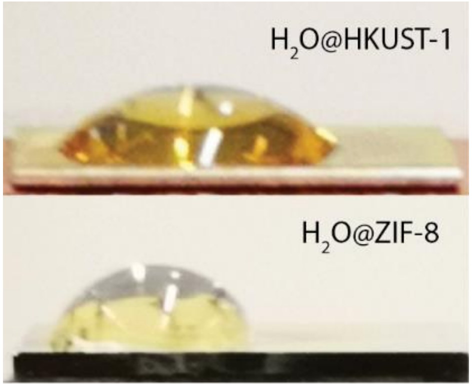

Figure S6: Rudimentary contact angle measurements showing hydrophilicity of the HKUST-1 SURMOF (top) and hydrophobicity of the ZIF-8 SURZIF (bottom).

Drops of water were deposited on HKUST-1 SURMOF and SURZIF-8. These measurements show the hydrophilic nature of HKUST-1 and the hydrophobic nature of ZIF-8.

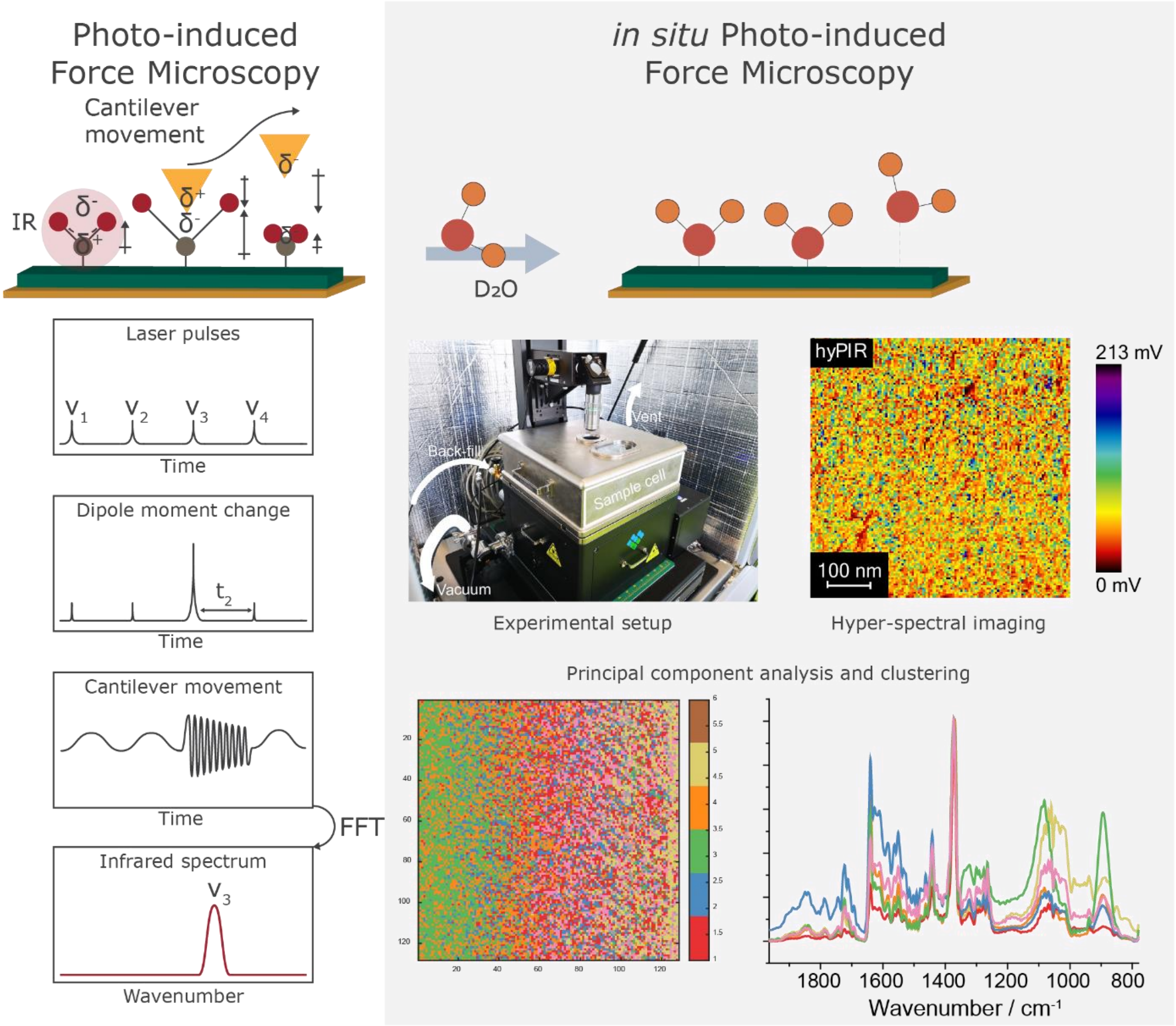

Figure S7: Experimental procedure for (in situ) Photo-induced Force Microscopy measurements. The PiFM technique uses a modulated laser to induce attractive forces between tip and sample. IR light is absorbed by bonds in the sample, which results in a change in the dipole moment of the bond. The gold-coated PiFM tip possesses a high polarizability and can therefore mirror this change in dipole moment. This mirroring of dipole moment change gives a rapid attractive force between tip and sample which leads to cantilever resonance. This tip resonance can be converted to a peak in the IR spectrum using a Fast-Fourier Transform (FFT). For in situ measurements, the PiFM tool was adapted to accommodate gas flows. During gas flow experiments, hyperspectral images, consisting of a full IR spectrum on each AFM pixel, were taken. These images were processed using Principal Component Analysis (PCA) and clustering, resulting in images clustered by spectral similarities.

Near-field infrared spectroscopy is a technology still in its early days. As a result, there are currently two atomic force microscopy (AFM)-IR techniques relying on similar, but distinct detection mechanisms: the photothermal AFM-IR (PT-AFM-IR) technique[21,22] and the Photo-induced Force Microscopy (PiFM).[12,23] The photothermal AFM-IR (PT-AFM-IR) technique monitors the thermal expansion of material upon IR light absorption, while the Photo-induced Force Microscopy (PiFM) technique detects IR light absorption by mirroring the change in the dipole moment of IR active bonds in its gold-coated AFM tip.

Both techniques couple AFM with a modulated (or pulsed) mid-IR laser. The required power setting of the laser (and several other parameters), however, are dependent on the phenomenon it aims to probe. In the case of the PiFM technique, a pulse of IR light (when absorbed by a bond) will lead to a change in the dipole moment of the probed bonds which is detected by a gold-coated AFM tip. The coating layer of gold possesses high polarizability and is therefore able to mirror the change in dipole moment occurring directly below the tip. A rapidly photo-induced change in attractive forces between

the tip and the sample is then detected and converted to IR signal. The required power is typically low for this technique.

In the case of PT-AFM-IR, a pulse of IR light (when absorbed by a bond) will lead to the rapid photothermal expansion of the material due to the induced vibrations. How much the material expands depends both on the used power (i.e. change in temperature), and on the thermal expansion coefficient according to the relation $\Delta V \sim \alpha \Delta T$, where ΔV is the change in volume, α is the thermal expansion coefficient and ΔT is the change in temperature. The thermal expansion coefficient is a material-inherent property, and organic materials typically possess a larger thermal expansion coefficient than inorganic materials. Since the technique requires a sufficiently large temperature increase to gain a detectable increase in volume, higher power levels are typically necessary.

Some debate has arisen, however, on the exact origins of the photo-induced force in PiFM. Overall, it was found that the main force giving rise to PiFM signal is due to dipole-dipole interaction mirroring, [12]yet some literature has suggested that in PiFM, multiple forces can give signal contributions. For example, there are reports that show that in order to explain the spectral profile of the photo-induced force, photothermal expansion has to be added as a contributing repulsive force.[24,25] However, a recent report suggests that this photothermal force has a negligible effect on the signal, but suggests opto-mechanical damping of the cantilever oscillation can explain the contrast mechanism of PiFM.[26] While the exact mechanism is still under debate, literature does agree that PiFM is a highly sensitive tool that can measure infrared nano-spectroscopy with an unparalleled high spatial resolution.

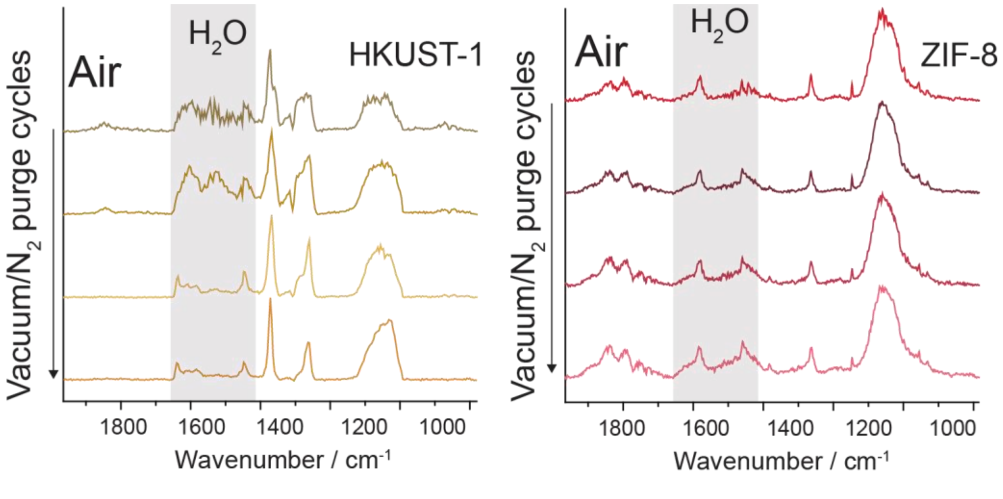

Figure S8: (left) PiFM spectra showing effective removal of water from HKUST-1 surface during the vacuum/N_2 purge cycling procedure. (right) PiFM spectra of the hydrophobic ZIF-8 are unaffected by the presence of H_2O.

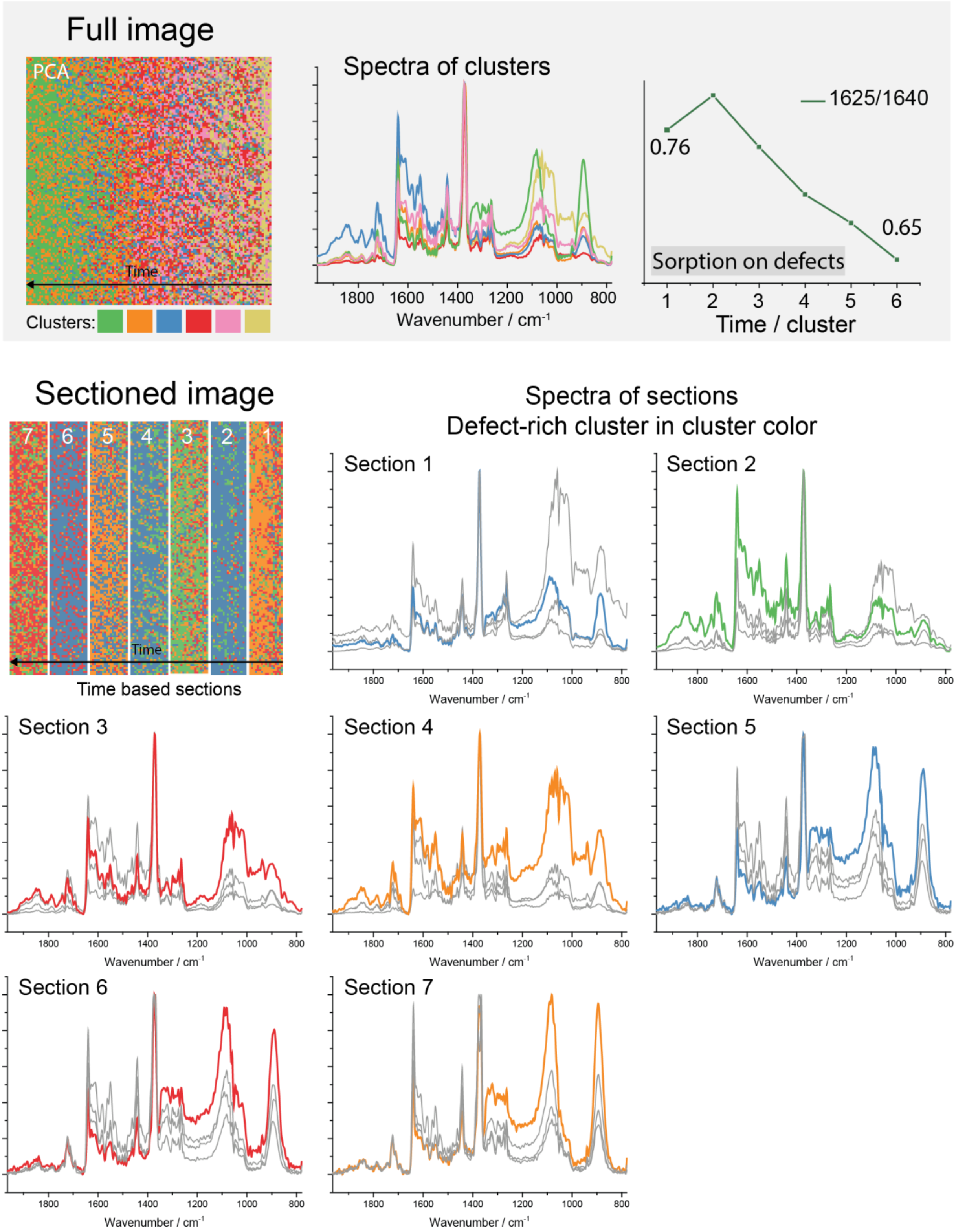

Figure S9: Principal Component Analysis (PCA) and clustering analysis of the hyperspectral (hyPIR) measurement of the HKUST-1 surface in D_2O flow. PCA and clustering was performed on the full hyPIR image to analyze sorption kinetics, as well as on concentration-dependent sections of the image, to analyze site-specific gas sorption isotherms. Spectra of clusters used for analysis are shown, colors correspond to clusters in images.

In PCA and clustering, spectra are clustered together based on spectral similarity, therefore pixels grouped together in a cluster show similar behavior during D_2O flow. Rather than using band ratio maps over the hyPIR measurement, we opted for using PCA and clustering to find ratios over clustered spectra to describe SURMOF

behavior in D_2O vapor. This was done as clustered spectra show an increased signal-to-noise ratio compared to showing ratios per single pixels. As a drawback, pixels no longer show individual behavior but belong to a cluster, and clustering parameters have to be carefully considered to verify that clustering is performed based on changes in ratios that are under investigation. However, a large advantage of PCA and clustering is that extreme outliers as a result of noise are prevented and therefore a more cohesive and easy to interpret result is presented.

The PCA image of the *in situ* hyPIR measurement shows a gradient over time, signaling a change in the spectrum over time (Fig. S9, top left/middle). This change is initiated by D_2O flow. We studied the averaged spectrum of the clusters used in PCA. In this analysis, cluster 1 (sand-colored) described the initial HKUST-1 in N_2, and cluster 6 (green) described HKUST-1 post-sorption. The clusters in between (2-5) describe the response to D_2O, where the lower the cluster number, the faster the response (e.g. pink). Of the spectra of these clusters, we calculated the ratio between a known defect vibration and the pristine paddlewheel vibration (Fig. S9, top right). This revealed that surface regions exhibiting a higher concentration of defects show a faster response to D_2O (Fig. S9, top right).

Then, we divided the clustered image into 7 time/concentration-dependent sections. This division into sections was done as it placed concentration constraints on the results. Since a gas sorption isotherm is a function of (D_2O) concentration, we used both the average spectrum of each section as well as PCA and clustering on each of the sections to accurately compare the isotherms. Each section of 18 x 128 pixels equaled 40 minutes of hyPIR measurement, thus corresponding of a time resolution of 1 second per pixel (Fig. S9, middle left). Of these sections we took the average spectrum (Fig. 4A) which we used for calculating the ratios depicting HKUST-1 behavior in the presence of D_2O sorption (Fig. 4B-D).

The creation of defects could be deduced from the PCA and clustering of the full image. However, again we wished to uncover chemical change as a result of defined increments of D_2O concentration. Therefore, each of these sections was segmented using PCA and clustering to identify pixels with high defect concentrations (1625/1640 cm^{-1}, spectra of defect rich clusters highlighted in Fig. S9) to show the development of defect sites over time within that D_2O concentration regime (Fig. 4F). Using the images showing defect generation, we were able to calculate the increase in density of defect sites over the surface as a function of D_2O concentration (Table S1).

Table S1: Table showing the increase in approximate defect site density of the HKUST-1 surface as a result of D_2O sorption. Percentages were calculated by taking the ratio between pixels of defect clusters and pixels of pristine clusters of the sectioned hyPIR image.

**Section / D_2O pressure**	**Defect cluster coverage percentage**
1 / 1500 ppm	2%
2 / 3000 ppm	9%
3 / 4500 ppm	7%
4 / 6000 ppm	18%
5 / 7500 ppm	21%
6 / 9000 ppm	23%
7 / 9000 ppm	25%

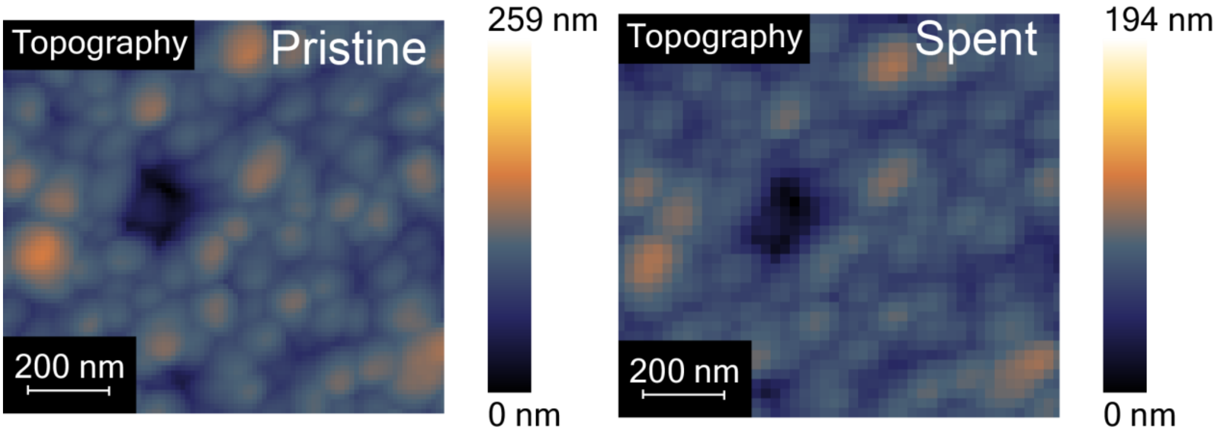

Figure S10: AFM images of the area used for the in-situ *PiFM measurement before- and after D_2O flow. Note that the spent image is taken in lower resolution (128x128 px instead of 256x256 px).*

In the AFM images, no structural changes are visible, yet PiFM showed strong chemical changes due to D_2O flow. This underlines the relevance of *in-situ* nano-spectroscopic tools for the analysis of SURMOFs during gas-sorption.

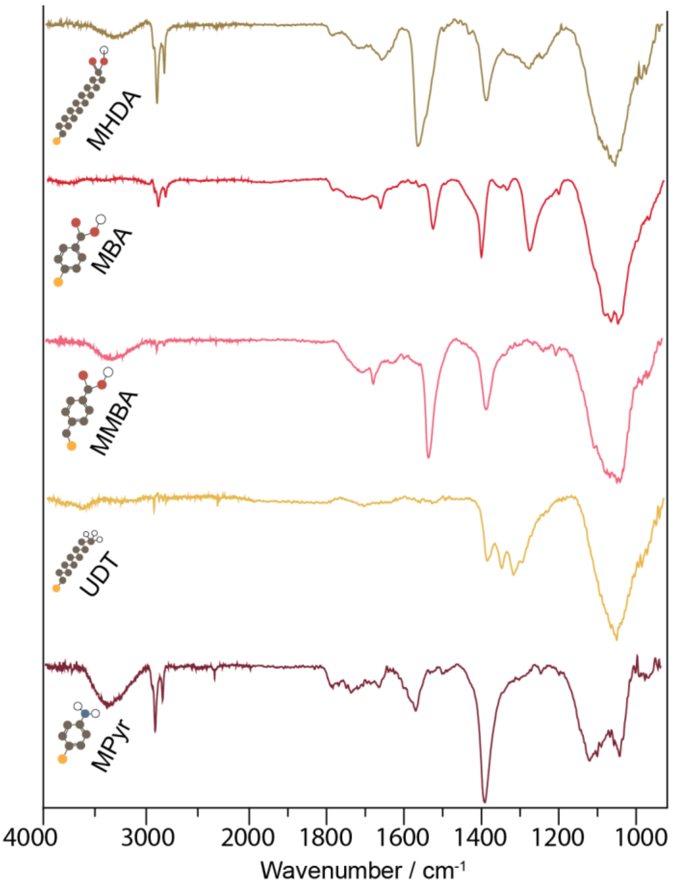

Figure S11: Full GI-ATR-IR spectra of the aliphatic and aromatic thiols used in the manuscript. Note the presence of a broad peak at 1050 cm^{-1} stemming from a germanium oxide layer on the GI-ATR crystal.

The spectra show the adsorption of the various thiols. Although it is very difficult to track any sulfur-based vibrations (S-H, C-S) that may hint towards Au-S bond formation, some film quality estimations of the -COOH thiols could be deduced from the ratio between C=O monomer vibrations (1750 cm^{-1}) and C=O dimer vibrations(1720-1706 cm^{-1}). The spectra were used to identify individual species in PiFM analysis.

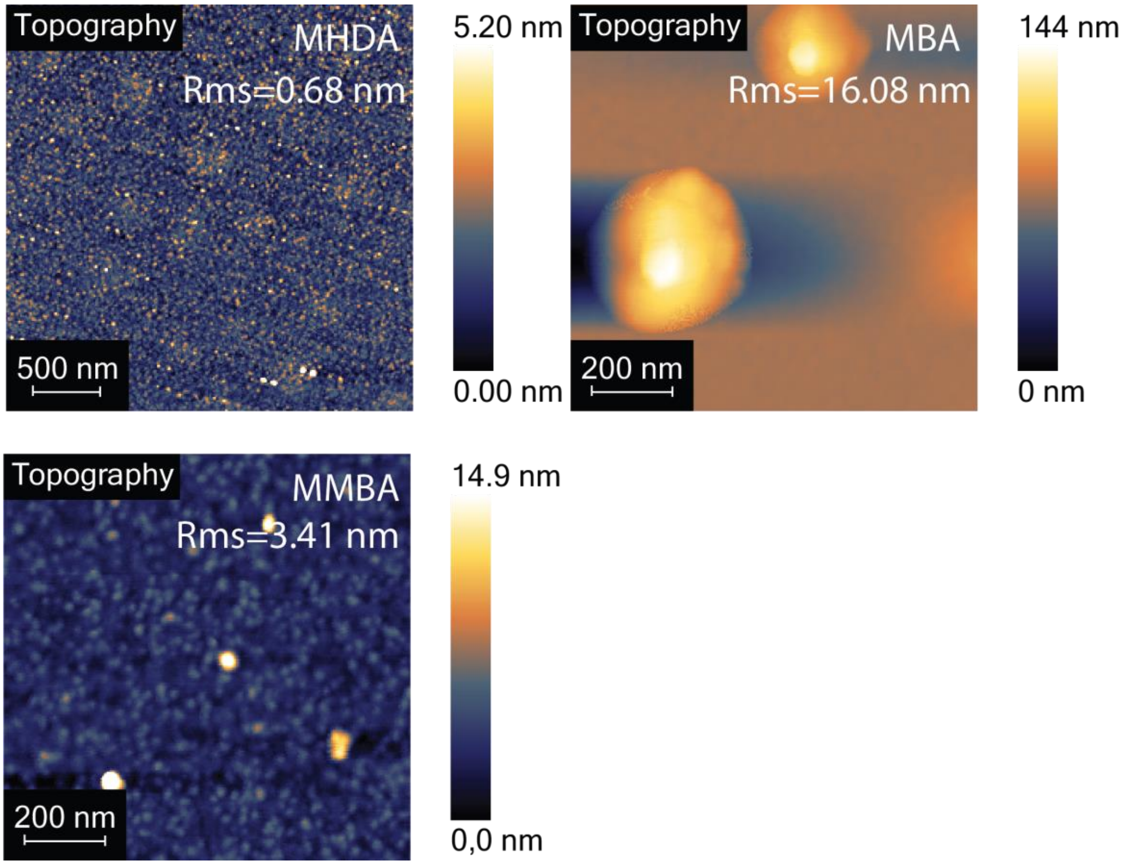

Figure S12: AFM images of the -COOH thiols MHDA, MBA, and MMBA. Aliphatic SAMs, such as MHDA, typically display neat Van der Waals-based stacking resulting in a homogeneous self-assembled monolayer displaying low surface roughness. Higher surface roughness was found for aromatic thiol molecules due to π-stacking interactions.

The long aliphatic chains of MHDA provide an excellent system for stacking in monolayer conformation (low rms value) on the surface through Van der Waals forces. The aromatic MBA thiol does not possess a long chain and stacks primarily through π-stacking which resulted in non-monolayer stacking. The heights of the observed features suggest that rather than just bilayer stacking, aggregation of thiols occurred. By introducing an additional carbon atom in the aromatic backbone, the MMBA molecule gained flexibility which allowed it to stack in semi-monolayer conformation. This is reflected in the low rms value for the MMBA surface.

References

[1] G. Delen, Z. Ristanović, L. D. B. Mandemaker, B. M. Weckhuysen, *Chem. - A Eur. J.* **2018**, *24*, 187–195.

[2] Y. Liu, F. Meirer, P. A. Williams, J. Wang, J. C. Andrews, P. Pianetta, *J. Synchrotron Radiat.* **2012**, *19*, 281–287.

[3] C. D. Bain, G. M. Whitesides, *J. Am. Chem. Soc.* **2002**, *110*, 6560–6561.

[4] C. D. Bain, J. Evall, G. M. Whitesides, *J. Am. Chem. Soc.* **2002**, *111*, 7155–7164.

[5] D. Fischer, A. Curioni, W. Andreoni, *Langmuir* **2003**, *19*, 3567–3571.

[6] S. J. Stranick, A. N. Parikh, Y.-T. Tao, D. L. Allara, P. S. Weiss, *J. Phys. Chem.* **2002**, *98*, 7636–7646.

[7] P. D. Jadzinsky, G. Calero, C. J. Ackerson, D. A. Bushnell, R. D. Kornberg, *Science (80-.).* **2007**, *318*, 430–433.

[8] L. A. Bumm, J. J. Arnold, L. F. Charles, T. D. Dunbar, D. L. Allara, P. S. Weiss, *J. Am. Chem. Soc.* **1999**, *121*, 8017–8021.

[9] W. Schrimpf, J. Jiang, Z. Ji, P. Hirschle, D. C. Lamb, O. M. Yaghi, S. Wuttke, *Nat. Commun.* **2018**, *9*, 1647.

[10] P. E. Laibinis, R. G. Nuzzo, G. M. Whitesides, *J. Phys. Chem.* **2002**, *96*, 5097–5105.

[11] Y.-J. Liu, N. M. Navasero, H.-Z. Yu, *Langmuir* **2004**, *20*, 4039–4050.

[12] D. Nowak, W. Morrison, H. K. Wickramasinghe, J. Jahng, E. Potma, L. Wan, R. Ruiz, T. R. Albrecht, K. Schmidt, J. Frommer, D. P. Sanders, S. Park, *Sci. Adv.* **2016**, *2*, e1501571.

[13] J. Löfgren, H. Grönbeck, K. Moth-Poulsen, P. Erhart, *J. Phys. Chem. C* **2016**, *120*, 12059–12067.

[14] H. Grönbeck, A. Curioni, W. Andreoni, *J. Am. Chem. Soc.* **2000**, *122*, 3839–3842.

[15] L. D. B. Mandemaker, M. Filez, G. Delen, H. Tan, X. Zhang, D. Lohse, B. M. Weckhuysen, *J. Phys. Chem. Lett.* **2018**, *9*, 1838–1844.

[16] O. Shekhah, M. Eddaoudi, *Chem. Commun.* **2013**, *49*, 10079.

[17] V. Chernikova, O. Shekhah, M. Eddaoudi, *ACS Appl. Mater. Interfaces* **2016**, *8*, 20459–20464.

[18] E. Biemmi, C. Scherb, T. Bein, *J. Am. Chem. Soc.* **2007**, *129*, 8054–8055.

[19] E. A. Ramírez, E. Cortés, A. A. Rubert, P. Carro, G. Benítez, M. E. Vela, R. C. Salvarezza, *Langmuir* **2012**, *28*, 6839–6847.

[20] Z. Ma, F. Rissner, L. Wang, G. Heimel, Q. Li, Z. Shuai, E. Zojer, *Phys. Chem. Chem. Phys.* **2011**, *13*, 9747–9760.

[21] A. Dazzi, R. Prazeres, F. Glotin, J. M. Ortega, *Opt. Lett.* **2005**, *30*, 2388–2390.

[22] A. Dazzi, C. B. Prater, *Chem. Rev.* **2017**, *117*, 5146–5173.

[23] J. Jahng, D. A. Fishman, S. Park, D. B. Nowak, W. A. Morrison, H. Kumar Wickramasinghe, E. O. Potma, *Acc. Chem. Res.* **2015**, *48*, 2671–2679.

[24] L. Wang, H. Wang, D. Vezenov, X. G. Xu, *J. Phys. Chem. C* **2018**, *122*, 23808–23813.

[25] J. Jahng, E. O. Potma, E. Seong Lee, *Anal. Chem.* **2018**, *90*, 11054–11061.

[26] M. A. Almajhadi, S. M. A. Uddin, H. K. Wickramasinghe, in *Conf. Lasers Electro-Optics*, Optical Society Of America, Washington, DC, **2020**, p. SW3G.7.
